# Supplementary material for: Delineating maternal influence in regulation of variance in major economic traits of White Leghorns: Bayesian insights
Source: PLoS One. 2024 Jul 26;19(7):e0307987. doi: 10.1371/journal.pone.0307987 (PMC11280281; doi:10.1371/journal.pone.0307987)
Supplement: Attachment — Submitted filename: PONE-D-24-11008_reviewer.pdf [file pone.0307987.s001.pdf]

PLOS ONE

Delineating maternal influence in regulation of variance in major economic traits of White Leghorns: Bayesian insights

--Manuscript Draft--

**Manuscript Number:**	PONE-D-24-11008
**Article Type:**	Research Article
**Full Title:**	Delineating maternal influence in regulation of variance in major economic traits of White Leghorns: Bayesian insights
**Short Title:**	Effect of maternal inheritance in on genetic parameters in chicken
**Corresponding Author:**	U. Rajkumar<br>Directorate of Poultry Research<br>Hyderabad, Telangana INDIA
**Keywords:**	Maternal effects; Genetic parameters; Variance; Bayesian analysis
**Abstract:**	Proper variance partitioning and estimation of genetic parameters at appropriate time interval is crucial for understanding the dynamics of trait variance and genetic correlations and for deciding the future breeding strategy of the population. This study was conducted on the same premise to estimate genetic parameters of major economic traits in a White Leghorn strain IWH using Bayesian methods and to identify the role of maternal effects in the regulation of trait variance. Three different models incorporating the direct additive effect (Model 1), direct additive and maternal genetic effect (Model 2) and direct additive, maternal genetic and maternal permanent environmental effects (Model 3) were tried to estimate the genetic parameters for body weight traits (birth weight, body weight at 16, 20, 40 and 52 weeks), Age at sexual maturity (ASM), egg production traits (egg production up to 24, 28, 40, 52, 64 and 72 weeks) and egg weight traits (egg weight at 28, 40 and 52 weeks). Model 2 and Model 3 with maternal effects were found to be the best having the highest accuracy for almost all the traits. The direct additive genetic heritability was moderate for ASM, moderate to high for body weight traits and egg weight traits and low to moderate for egg production traits. Though the maternal heritability (h2mat) and permanent environmental effect (c2mpe) was low (<0.1) for most of the traits, they formed an important component of trait variance. Traits like egg weight at 28 weeks (0.14±0.06) and egg production at 72 weeks (0.13±0.07) reported comparatively higher values for c2mpe and h2mat respectively. Additive genetic correlation was high and positive between body weight traits, between egg weight traits, between consecutive egg production traits and between body weight and egg weight traits. However, a negative genetic correlation existed between egg production and egg weight traits, egg production and body weight traits, ASM and early egg production traits. Overall, a moderate positive genetic correlation was estimated between ASM and body weight traits and ASM and egg weight traits. Based on our findings, we can deduce that maternal effects constitute an important source of variation for all the major economic traits in White Leghorn and should be necessarily considered in genetic evaluation programs.
**Order of Authors:**	Aneet Kour, PhD
	R N Chatterjee, PhD
	K S Rajaravindra, PhD
	L Leslie Leo Prince
	Santosh Haunshi
	M Niranjan
	B L N Reddy
	U. Rajkumar
**Additional Information:**	
**Question**	**Response**

Powered by Editorial Manager® and ProduXion Manager® from Aries Systems Corporation

**Financial Disclosure**<br><br>Enter a financial disclosure statement that describes the sources of funding for the work included in this submission. Review the submission guidelines for detailed requirements. View published research articles from *PLOS ONE* for specific examples.<br><br>This statement is required for submission and **will appear in the published article** if the submission is accepted. Please make sure it is accurate.<br><br>**Funded studies**<br>Enter a statement with the following details:<br>• Initials of the authors who received each award<br>• Grant numbers awarded to each author<br>• The full name of each funder<br>• URL of each funder website<br>• Did the sponsors or funders play any role in the study design, data collection and analysis, decision to publish, or preparation of the manuscript?<br><br>Did you receive funding for this work?	The author(s) received no specific funding for this work.
**Competing Interests**<br><br>Use the instructions below to enter a competing interest statement for this submission. On behalf of all authors, disclose any competing interests that could be perceived to bias this work—acknowledging all financial support and any other relevant financial or non-financial competing interests.<br><br>This statement is required for submission and **will appear in the published article** if the submission is accepted. Please make sure it is accurate and that any funding sources listed in your Funding Information later in the submission form are also declared in your Financial Disclosure statement.	The authors do not have have any competing interests

View published research articles from *PLOS ONE* for specific examples.<br><br>**NO authors have competing interests**<br><br>Enter: *The authors have declared that no competing interests exist.*<br><br>**Authors with competing interests**<br><br>Enter competing interest details beginning with this statement:<br><br>*I have read the journal's policy and the authors of this manuscript have the following competing interests: [insert competing interests here]*<br><br>* typeset	
**Ethics Statement**<br><br>Enter an ethics statement for this submission. This statement is required if the study involved:<br><br>• Human participants<br>• Human specimens or tissue<br>• Vertebrate animals or cephalopods<br>• Vertebrate embryos or tissues<br>• Field research<br><br>Write "N/A" if the submission does not require an ethics statement.<br><br>General guidance is provided below. Consult the submission guidelines for detailed instructions. **Make sure that all information entered here is included in the Methods section of the manuscript.**	The study was approved by Institutional Animal Ethics Committee of ICAR-DPR vide IAEC/DPR/20/7.

Powered by Editorial Manager® and ProduXion Manager® from Aries Systems Corporation

**Format for specific study types**<br><br>**Human Subject Research (involving human participants and/or tissue)**<br>• Give the name of the institutional review board or ethics committee that approved the study<br>• Include the approval number and/or a statement indicating approval of this research<br>• Indicate the form of consent obtained (written/oral) or the reason that consent was not obtained (e.g. the data were analyzed anonymously)<br><br>**Animal Research (involving vertebrate animals, embryos or tissues)**<br>• Provide the name of the Institutional Animal Care and Use Committee (IACUC) or other relevant ethics board that reviewed the study protocol, and indicate whether they approved this research or granted a formal waiver of ethical approval<br>• Include an approval number if one was obtained<br>• If the study involved *non-human primates*, add *additional details* about animal welfare and steps taken to ameliorate suffering<br>• If anesthesia, euthanasia, or any kind of animal sacrifice is part of the study, include briefly which substances and/or methods were applied<br><br>**Field Research**<br><br>Include the following details if this study involves the collection of plant, animal, or other materials from a natural setting:<br>• Field permit number<br>• Name of the institution or relevant body that granted permission	
**Data Availability**<br><br>Authors are required to make all data underlying the findings described fully available, without restriction, and from the time of publication. PLOS allows rare exceptions to address legal and ethical concerns. See the PLOS Data Policy and FAQ for detailed information.	No - some restrictions will apply

Powered by Editorial Manager® and ProduXion Manager® from Aries Systems Corporation

A Data Availability Statement describing where the data can be found is required at submission. Your answers to this question constitute the Data Availability Statement and **will be published in the article**, if accepted.<br><br>**Important:** Stating 'data available on request from the author' is not sufficient. If your data are only available upon request, select 'No' for the first question and explain your exceptional situation in the text box.<br><br>Do the authors confirm that all data underlying the findings described in their manuscript are fully available without restriction?	
**Describe where the data may be found in full sentences. If you are copying our sample text, replace any instances of XXX with the appropriate details.**<br><br>• If the data are **held or will be held in a public repository**, include URLs, accession numbers or DOIs. If this information will only be available after acceptance, indicate this by ticking the box below. For example: *All XXX files are available from the XXX database (accession number(s) XXX, XXX.*).<br>• If the data are all contained **within the manuscript and/or Supporting Information files**, enter the following: *All relevant data are within the manuscript and its Supporting Information files.*<br>• If neither of these applies but you are able to provide **details of access elsewhere**, with or without limitations, please do so. For example:<br><br>*Data cannot be shared publicly because of [XXX]. Data are available from the XXX Institutional Data Access / Ethics Committee (contact via XXX) for researchers who meet the criteria for access to confidential data.*<br><br>*The data underlying the results presented in the study are available from (include the name of the third party*	The data is available with ICAR-DPR, Hyderabad repository, which will be shared on request.

Powered by Editorial Manager® and ProduXion Manager® from Aries Systems Corporation

and contact information or URL).<br>• This text is appropriate if the data are owned by a third party and authors do not have permission to share the data.<br><br>* typeset	
Additional data availability information:	Tick here if the URLs/accession numbers/DOIs will be available only after acceptance of the manuscript for publication so that we can ensure their inclusion before publication.

Powered by Editorial Manager® and ProduXion Manager® from Aries Systems Corporation

Manuscript Click here to access/download;Manuscript;AK_19-03-2024.docx

Delineating maternal influence in regulation of variance in major economic traits of White Leghorns: Bayesian insights

Aneet Kour*, R.N. Chatterjee K.S. Rajaravindra, L. Leslie Leo Prince, Santosh Haunshi, M. Niranjan, B.L.N. Reddy and U. Rajkumar*

Poultry Genetics and Breeding Division

ICAR-Directorate of Poultry Research, Rajendranagar, Hyderabad, Telangana – 500030

*Corresponding author

Corresponding authors email id: ANEET.KOUR@icar.gov.in; ullengala@yahoo.com

Abstract

Proper variance partitioning and estimation of genetic parameters at appropriate time interval is crucial for understanding the dynamics of trait variance and genetic correlations and for deciding the future breeding strategy of the population. This study was conducted on the same premise to estimate genetic parameters of major economic traits in a White Leghorn strain IWH using Bayesian methods and to identify the role of maternal effects in the regulation of trait variance. Three different models incorporating the direct additive effect (Model 1), direct additive and maternal genetic effect (Model 2) and direct additive, maternal genetic and maternal permanent environmental effects (Model 3) were tried to estimate the genetic parameters for body weight traits (birth weight, body weight at 16, 20, 40 and 52 weeks), Age at sexual maturity (ASM), egg production traits (egg production up to 24, 28, 40, 52, 64 and 72 weeks) and egg weight traits (egg weight at 28, 40 and 52 weeks). Model 2 and Model 3 with maternal effects were found to be the best having the highest accuracy for almost all the traits. The direct additive genetic heritability was moderate for ASM, moderate to high for body weight traits and egg weight traits and low to moderate for egg production traits. Though the maternal heritability (h^2_{mat}) and permanent environmental effect (c^2_{mpe}) was low (<0.1) for most of the traits, they formed an important component of trait variance. Traits like egg weight at 28 weeks (0.14±0.06) and egg production at 72 weeks (0.13±0.07) reported comparatively higher values for c^2_{mpe} and h^2_{mat} respectively. Additive genetic correlation was high and positive between body weight traits, between egg weight traits, between consecutive egg production traits and between body weight and egg weight traits. However, a negative genetic correlation existed between egg production and egg weight traits, egg production and body weight traits, ASM and early egg production traits. Overall, a moderate positive genetic correlation was estimated between ASM and body weight traits and ASM and egg

weight traits. Based on our findings, we can deduce that maternal effects constitute an important source of variation for all the major economic traits in White Leghorn and should be necessarily considered in genetic evaluation programs.

Introduction

Intense selection for higher genetic gain is the hallmark of poultry breeding programs throughout the world [1-2]. It causes negative linkage disequilibrium between the loci influencing the trait and hence, leads to a reduction in the additive genetic variance (Bulmer effect) for the trait under selection [3-4]. This further gets aggravated in case of small and closed populations (like inbred lines) where strong directional selection in a trait over the generations can reduce the additive component of variance substantially and effectively leaves little for selection to act upon [5]. Reduction in additive genetic variance will invariably affect the trait heritability and estimated breeding values (EBVs) of the individuals for the trait [6]. Furthermore, in case of multi-trait selection, long-term selection can even change the genetic correlations between the traits due to fixation of positively pleiotropic genes in the population and dominance of negative pleiotropy [4]. Therefore, computation and monitoring of genetic parameters of a population from time to time is crucial to study the dynamics of genetic variance and to obtain appropriate selection response. It also helps in optimization of breeding programs by indicating whether there is a need for introduction of genetic variation from outside [7].

Accuracy of the estimated genetic parameters is contingent on the proper variance partitioning for the trait under consideration [8-9]. The true picture of the additive genetic worth of a population for a trait will come to light once the variance attributable to every possible source of variation *viz*. maternal genetic or indirect genetic effect and maternal permanent environment is

properly accounted for in the model [10]. Ignoring these effects in variance estimation can produce seriously biased estimates and can adversely affect the genetic improvement program [11-13]. Moreover, in poultry, many of the traits like age at first egg, birth weight and early body weights and egg weights etc. are maternally influenced traits i.e. there is a low to moderate maternal heritability component involved in the total trait variance [14-17]. Sometimes, the maternal variance component for the traits even exceeds the direct additive component [18]. Therefore, proper partitioning of a trait variance can minimize uncertainty or error variance and prevents the overestimation of additive genetic component [19-20].

Another important factor influencing the accuracy of genetic parameters is the sample size. Larger the sample size, more precise will be the genetic estimates [21-22]. Whenever sample size is less, the traditional likelihood-based analysis like REML (Restricted Maximum Likelihood) can give rise to biased genetic estimates [23]. In such cases, Bayesian sampling can more effectively model data with smaller effective population sizes given that proper priors are specified [24]. In Bayesian approach, prior information is effectively integrated with the data likelihood to create a posterior distribution of samples from which inferences are drawn [25]. Thus, as compared to the REML approach, it improves the accuracy of genetic estimates obtained from small populations. These days, Bayesian methods are being increasingly used to derive accurate genetic estimates in native chicken breeds as well as in commercial lines [26-28]. In India, several studies carried out in the past have dealt with variance partitioning into maternal genetic and permanent environmental components in chickens [9-10, 20]. However, to the best of our knowledge, none of the studies carried out in chicken populations of India have used Bayesian inferences for obtaining genetic estimates. Therefore, this study was conceived to appropriately partition the

variance components and estimate genetic parameters in a population of Indian White Leghorn by trying different BLUP animal models using Bayesian approach.

Materials and Methods

Study details

The present investigation was performed in the IWH line of White Leghorn layer breed maintained at ICAR-Directorate of Poultry Research, Hyderabad, Telangana, India. This line has been developed by selecting parents for higher egg production up to 64 weeks of age using Osborne Index. A total of 1434 chicks belonging to three (3) generations (2020-23) and five (5) hatches were considered for this study. In each generation, number of sires and dams used for regeneration were around 50 and 250 respectively. Chicks were wing banded at the time of pedigree hatch and reared in a deep litter brooder house till they attained 16 weeks of age. At six weeks, sexing was done to separate males and females. Layer starter diet (0-6 weeks) based on maize and soybean meal with 18% crude protein and 2800 kcal/kg ME was fed *ad libitum* to the chicks up to 16 weeks of age. Thereafter, layer grower ration was fed from 6-16 weeks of age which comprised of 18% crude protein and 2700 kcal/kg ME. After 16 weeks, adult males and females were transferred to individual cages and were fed layer breeder ration with 16.5% crude protein, 2650 kcal/kg ME and 3.5% calcium. In the layer cage house, daily allowance of eight hours of day light and seven hours of artificial light was provided. Since the farm is situated in the Deccan plateau region of India at an elevation of 500 metres above mean sea level (geographical coordinates 17°23' N and 78° 28' E), the place experiences hot and humid tropical climate. Therefore, in order to avoid heat stress during summers, ambient temperature was maintained in the layer house through sprinklers installed on roof top. Also, a standard vaccination schedule was followed in the farm and birds were vaccinated against all the major diseases: Marek's disease (Day 1), New Castle Disease,

Lasota (Day 6 and Day 28), Infectious Bursal Disease (Day 14 and Day 24), Fowl Pox (Day 42), New Castle Disease, R_2B (Day 65), Infectious Coryza (Day 84) and Triple killed vaccine (Infectious Bursal Disease, Infectious Bronchitis and New Castle Disease inactivated) (112th day and 45th week). The recorded mortality in the flock was less than 5%.

The study was approved by Institutional Animal Ethics Committee of ICAR-DPR vide IAEC/DPR/20/7.

Traits recorded

Data spanning three generations from 2020-23 was recorded for different production and growth traits up to 72 weeks of age in the female IWH population. First of all, body weight of chicks at the time of hatch (BW0) was recorded. Thereafter, when female birds were housed in individual cages, body weight at different intervals *viz*. 16 (BW16), 20 (BW20), 40 (BW40) and 52 (BW52) weeks was successively recorded. Body weights were measured to 0.1 g accuracy using a digital weighing balance. Reproduction trait like age at sexual maturity (ASM) was also recorded for all the birds.

Production data comprised of egg production or egg numbers up to 24 (EP24), 28 (EP28), 40 (EP40), 52 (EP52), 64 (EP64) and 72 (EP72) weeks and egg weights at 28 (EW28), 40 (EW40) and 52 (EW52) weeks of age. Egg production of individual birds was recorded daily and egg production up to a certain age was arrived at by summing the daily production up to that age. Egg production data after 40 weeks was not available for the third generation. Therefore, EP52, EP64 and EP72 data used to derive the genetic parameter estimates belonged to two generations only.

Further, egg weight at a particular age was estimated by taking the average of egg weight measurements of five consecutive days at that age. For instance, at 28 weeks of age, egg weight

was recorded for five successive days for each bird and the average of those five measurements was finally considered as EW28.

Recorded data was thoroughly screened for the removal of outliers and observations falling within Mean ± 3S.D. of normal distribution were retained for further analysis. The data structure of the study population for the traits has been presented in Table 1.

Table 1: Pedigree and data summary of the study population

Trait	No. of birds with records	No. of sires	No. of dams	Mean±S.E.	S.D.	C.V. (%)
**ASM**	1319	117	347	137.56±0.27	8.94	6.50
**BW0**	936	88	220	35.05±0.11	3.35	9.56
**BW16**	1064	118	340	973.31±3.83	124.61	12.80
**BW20**	1145	72	223	1217.23±4.15	140.32	11.53
**BW40**	995	115	334	1480.83±6.15	194.06	13.10
**BW52**	709	71	215	1516.04±7.13	189.92	12.53
**EW28**	1041	116	336	47.77±0.09	3.06	6.41
**EW40**	937	115	329	51.34±0.11	3.34	6.50
**EW52**	662	71	213	54.17±0.16	4.16	7.68
**EP24**	1051	106	318	26.13±0.23	7.19	27.54
**EP28**	1046	110	324	49.11±0.33	10.54	21.46
**EP40**	1021	110	320	117.56±0.58	18.64	15.86
**EP52**	669	72	193	188.59±0.77	19.91	10.56
**EP64**	603	70	189	251.70±1.04	25.57	10.16

**EP72**	546	69	180	283.49±1.26	29.43	10.38

Statistical analysis

Generation (N = 3) and hatch (N = 5) were identified as the non-genetic (fixed) factors influencing the traits. Three BLUP animal models incorporating one or more of the variance components (direct additive, maternal genetic and maternal permanent environmental effects) along with fixed effects and residual were tried to identify the best fitted model for a trait.

The linear mixed models used are as follows:

<u>Model 1</u>: **y = Xb + Zu + e**

where y = a vector of observations (or records) for the trait, b = vector of fixed effects for the trait, u = vector of random additive genetic effect for the trait, e = vector of random residual effects, X = incidence matrix relating observations to fixed effects, Z = incidence matrix relating records to random animal effects.

Here, $var(u) = A\sigma^2_u = G$

$var(e) = I\sigma^2_e = R$

where A=Numerator relationship matrix, I = Identity matrix

In this case, variance for a trait is given by: var(y) = var(Zu + e)

$$var(y) = ZGZ' + R$$

$$var(y) = ZGZ' + I\sigma^2_e$$

<u>Model 2</u>: **y = Xb + Zu + Wd + e**

where d = vector of random maternal (indirect) genetic effect, W = incidence matrix relating records to maternal genetic effects; rest of the notations same as mentioned above.

Since there is covariance between additive genetic effect and maternal genetic effect, the combined variance for both the effects is:

$$\mathrm{var}\begin{bmatrix} u \\ d \end{bmatrix} = [gA]$$

where, $g = \begin{pmatrix} g_{11} & g_{12} \\ g_{21} & g_{22} \end{pmatrix}$

g_{11} = additive genetic variance for direct effects, g_{22} = additive genetic variance for maternal effects, g_{12} = additive genetic covariance between direct and maternal effects

In this case, variance for a trait is given by:

$$\mathrm{var}(y) = [Z\ W] \begin{pmatrix} g_{11}A & g_{12}A \\ g_{21}A & g_{22}A \end{pmatrix} \begin{pmatrix} Z' \\ W' \end{pmatrix} + I\sigma^2_e$$

<u>Model 3</u>: **y = Xb + Zu + Wd + Mpe + e**

where pe = vector of random maternal permanent environmental effect, M = incidence matrix relating records to maternal permanent environmental effect, rest of the notations same as mentioned above

Here, $\mathrm{var}(pe) = I\sigma^2_{pe}$

In this case, variance for a trait is given by:

$$\mathrm{var}(y) = [Z\ W] \begin{pmatrix} g_{11}A & g_{12}A \\ g_{21}A & g_{22}A \end{pmatrix} \begin{pmatrix} Z' \\ W' \end{pmatrix} + MI\sigma^2_{pe}M' + I\sigma^2_e$$

The above mentioned animal models were analyzed using BLUPF90 family of programs [29]. First of all, renumbering program RENUMF90 was used for data quality checking and

obtaining basic statistics. This program processed the input and created new data, pedigree and parameter files for further analysis. Subsequently, GIBBSF90+ program was executed to obtain marginal posterior distribution for each parameter in the model. Additional interactive inputs included: the chain was run for 2,00,000 iterations with 2000 as burn-in and 50 as thinning interval. This effectively means that a Gibbs chain was run 2,00,000 times with the first 2000 samples discarded and thereafter, one in every 50th sample was stored. Further, POSTGIBBSF90 program was run to analyze the stored samples and obtain posterior means, posterior standard deviations and highest posterior density interval (HPDI) for the genetic parameters for each trait. Convergence of the chain generated by Gibbs sampler was diagnosed based on summary parameters like autocorrelation between lags, effective sample size and tests like Geweke diagnostic test [30]. This was further verified by plotting trace plots for the parameters in order to rule out the existence of any trend in the estimates.

Since three different models were analyzed for each trait, the model with the lowest DIC (Deviance Information Criterion) was selected as the best one appropriately partitioning the existing variance of the concerned trait [31]. Posterior estimates of (co)variance, heritability and other genetic parameters were deduced based on the best fitted model.

Results and Discussion

Partitioning of variance and posterior heritability estimates

The posterior estimates of (co)variance and heritability for the traits using different models are presented in Table 2 and 3. Based on the findings, it can be assumed that maternal genetic and permanent environmental effects of dam contribute an important source of variation in the population [32-33]. Maternal contribution in the expression of birth weight and early growth traits in the progeny has already been widely documented [16, 18, 34]. It is regulated partly by the

additive genetic component of the dam and partly by the permanent environment provided by the mother to her offspring [35]. The effect of maternal inheritance on egg sizes produced by the progeny has also been reported [36]. Maternal physiology and permanent environment can modulate offspring development and performance through egg traits like egg size and composition [14].

Age at sexual maturity

As in other livestock species, ASM is an important factor regulating egg production and fertility in chicken [37-38]. Also, since body weight crucially regulates the attainment of sexual maturity, ASM can be touted as a trait with important implications on almost all the major economic traits in layers [39]. The trait variation is partly regulated by genes and partly by environment in such a way that it is medium heritable [40]. This was further vindicated by our results with the direct additive h^2 for the trait in the best model being 0.32±0.13. Interestingly, in model 1, where only additive genetic variance was considered as a source of variation, h^2 for the trait was comparatively low (0.196±0.049). Inclusion of maternal sources of variation substantially improved the goodness of fit of the model and h^2 estimate, thus highlighting the importance of maternal inheritance in trait regulation [16].

Body weight traits

Body weight traits exert significant influence on the egg production performance and egg quality traits during the laying cycle [41-42]. Models incorporating either maternal genetic effect (Model 2) or maternal genetic and permanent environmental effects (Model 3) were the best fitted models for all the traits except BW0 or birth weight. This finding is quite contrary to the reported literature where birth weight has been known to be widely influenced by maternal effects in a way that chick birth weight is often analyzed as a maternal trait [43]. The direct additive heritability in

the best model (Model 1) for BW0 was quite high (0.73±0.09) whereas in the other two models, it was low (Model 2: 0.17±0.14)) to medium (Model 3: 0.22±0.14) heritable. This finding may be considered in the light of the fact that though DIC effectively balances model accuracy against complexity (determined by effective number of parameters in a model) by penalizing the loss function, some workers have reported better methods for Bayesian model comparisons [44-46]. Model 2 reported very high maternal heritability for the trait (0.51±0.14) which got partitioned between maternal genetic (0.29±0.16) and permanent environmental effects (0.18±0.10) in model 3. HPDI depicting the 95% credible interval in a posterior distribution was also high (0.55-0.89) for the trait.

Rest of the body weight traits also exhibited medium to high posterior heritability means with BW20 (0.44±0.15) and BW40 (0.56±0.16) reporting higher values. Chandan and co-workers [40] reported exactly similar h^2 estimate for BW20 (0.443±0.143) in the same layer line based on two generations data (2015-17) using REML approach. Almost similar h^2 estimates (0.45±0.13) for the trait were also reported by Manjeet and co-workers [47] in a synthetic white leghorn population from India. The estimated h^2 for the trait BW40 in our study is higher than those reported by a study for the trait in the same population [40]. This is in line with the fact that Bayesian analysis can improve the estimates by reducing the mean square error (due to prior incorporation), hence, it gives higher h^2 values when compared to REML analysis [48-49]. Nonetheless, most of the workers have also reported moderate to high heritability for BW40 in Indian White Leghorn synthetic populations [50-51]. The value of h^2 for BW16 (0.35±0.10) in our study was similar to that obtained by Rosa and coworkers [52] in a white leghorn population. The trait BW52 was also medium heritable (0.39±0.16) which is in line with the observations made by most of the earlier workers [53-54].

Based on our findings, there is enough additive genetic variation available in the body weight traits in IWH population which can be exploited (depending on the trait correlations) to achieve the desired effect in the primary trait of selection [55].

Egg weight traits

Egg weight plays an important role in deciding the economics of a laying hen and is known to be heavily influenced by egg quality traits like shell thickness, albumen height, albumen weight, yolk index etc. [56-57]. Based on our findings, the variance of all the egg weight traits can be partitioned into additive genetic, maternal genetic and permanent environmental effects as Model 3 was adjudged as the best fitted model for all these traits. EW28, in particular, showed maternal heritability of 0.14±0.06, which underlies the importance of maternal inheritance in regulating the trait variance. Maternal genetics and environment mediates offspring fitness through its effect on egg size and weight. Hence, maternal variance can be successfully exploited to improve egg weight in chickens [58-59].

Direct additive h^2 for EW28 was 0.33±0.12 which was in agreement with the earlier reports in the same population [59]. Chandan and co-workers [40] reported slightly higher value (0.36±0.10) of the trait in the study population. This indicates the effectiveness of our breeding program since adequate genetic variance for the trait has been maintained over the past several decades. As far as the trait EW40 was concerned, the estimates of h^2 reported by earlier studies were much higher than the present one. Although in our study, h^2 was 0.38±0.13, Chatterjee and co-workers [60] obtained an estimate of 0.54±0.19 while Chandan et al. [40] got h^2 value as 0.58±0.11. The reason could be that both the studies did not partition the maternal components of the trait variation. On the other hand, Veeramani et al. [61] reported a lower estimate of sire based h^2 for the trait (0.27±0.08) in IWN strain of White Leghorn population in India. It may be due to

the fact that sire model assumes genetic uniformity of the mates and hence, does not takes into account the additive genetic relationships between all the individuals in the population [62].

For EW52, while we obtained a very high posterior h^2 estimate (0.73±0.22) in IWH line, earlier reports on another White Leghorn line IWK reared in the same environment have obtained much lower estimates (0.18±0.08) [51]. The probable reason could be that IWK line has been selected for egg weight along with egg numbers due to which additive variance for the trait may have been exhausted due to selection [63].

Egg Production traits

Early production traits like EP24 showed medium heritability estimates (0.29±0.11) while rest of the egg production traits had lower heritabilities. This is in agreement with the earlier findings that h^2 in the initial phase of production cycle was relatively high [64-65]. Traits EP40 and EP52 signifying almost the middle phase of the laying cycle had similar heritabilities (0.19±0.10 and 0.19±0.13 respectively). It indicates the possibility of analyzing egg production by dividing into three *viz*., early, middle and later phases of laying cycle due to high genetic correlations and similar heritabilities between the consecutive traits [66].

Reported literature suggests that direct additive heritability is higher during the initial and later stages of production cycle and comparatively lesser during the peak production period or middle phase of laying cycle [64]. In our case, there is almost a linear declining trend of heritability across the laying cycle. EP64 and EP72 traits reported very low additive heritability with higher posterior standard deviations (0.05±0.05 and 0.09±0.07 respectively). The probable reason could be that studies reporting higher h^2 estimates in the later period of the laying cycle have used random regression models for fitting the egg production data. Random regression models are better suited for fitting longitudinal traits like egg production because they allow studying the trait variation as

a function of time [67]. Also, only two generations data was available with respect to the later traits like EP64 and EP72 which may have also reduced the precision of the estimates.

All egg production traits had somewhat maternal influence in their expression. For EP28, the role of maternal permanent environment in influencing egg production was very high (0.36±0.25). Traits EP40 and EP52 also showed similar partitioning of maternal h^2 attributing a value of 0.08±0.04 and 0.08±0.05 respectively to this component. Similarly, for EP64 and EP72 traits, maternal h^2 (0.09±0.05 and 0.13±0.07 respectively) was higher than the additive heritability estimates. This may appear on account of adaptive maternal effects which tend to improve the fitness of the offspring. Maternal environment can play a major role in the phenotype of offspring fitness by influencing the egg production and reproduction traits in incorporating data for more generations and increasing the sample size.

The complex interplay of direct additive, maternal genetic and maternal environmental variation in different economic traits of poultry can result in the final phenotypic expression of a trait and hence, all of these must be included in the prediction models [70].

Table 2: Posterior estimates of variance components and heritability for ASM and body weight traits using different models (σ^2_a: Additive genetic variance; σ^2_{mat}: maternal genetic variance; σ_{am}: covariance between additive genetic and maternal genetic effects; σ^2_{mpe}: maternal permanent environmental variance; $h^2_{additive}$ ± S.D.: Posterior direct additive heritability mean ± Posterior standard deviation; $HPDI_{additive}$: Highest Posterior Density Interval for heritability; h^2_{mat} ± S.D.: Posterior maternal heritability mean ± Posterior standard deviation; c^2_{mpe} ± S.D.: Posterior maternal permanent environmental effect mean ± Posterior standard deviation; DIC: Deviance Information Criterion)

**Parameters**	σ^2_a	σ^2_{mat}	σ_{am}	σ^2_{mpe}	σ^2_e	$h^2_{additive}$ ± S.D.	$HPDI_{additive}$	h^2_{mat} ± S.D.	c^2_{mpe} ± S.D.	DIC
**ASM**										
**Model 1**	15.64	-	-	-	63.76	0.196±0.049	0.104-0.296	-	-	7822.304
**Model 2**	26.51	7.16	-9.28	-	60.89	0.31±0.13	0.09-0.58	0.083±0.043	-	6900.79
**Model 3**	**27.26**	**5.87**	**-9.52**	**1.71**	**60.11**	**0.32±0.13**	**0.07-0.55**	**0.07±0.04**	**0.02±0.02**	**6898.27**
**BW0**										
**Model 1**	**8.59**	**-**	**-**	**-**	**3.17**	**0.73±0.09**	**0.55-0.89**	**-**	**-**	**4273.69**
**Model 2**	2.02	5.93	-2.08	-	5.83	0.17±0.14	0.008-0.44	0.51±0.14	-	4628.29
**Model 3**	2.58	3.44	-1.92	2.15	5.47	0.22±0.14	0.01-0.50	0.29±0.16	0.18±0.10	4597.47
**BW16**										
**Model 1**	3765.8	-	-	-	7797.8	0.32±0.005	0.22-0.44	-	-	12824.21
**Model 2**	**4091.7**	**612.25**	**-589.68**	**-**	**7570**	**0.35±0.10**	**0.16-0.55**	**0.05±0.04**	**-**	**12813.1**
**Model 3**	4020.4	609.25	-625	172.31	7545.5	0.34±0.11	0.14-0.54	0.052±0.03	0.02±0.01	12814.82
**BW20**										
**Model 1**	5567.5	-	-	-	9020.9	0.38±0.07	0.26-0.51	-	-	12544.52
**Model 2**	6139.5	1354.7	-1190.5	-	8484	0.41±0.13	0.18-0.67	0.09±0.05	-	12515.3
**Model 3**	**6569.5**	**1060.6**	**-1420.4**	**389.69**	**8250.5**	**0.44±0.15**	**0.16-0.74**	**0.07±0.04**	**0.03±0.02**	**12499.51**
**BW40**										
**Model 1**	16654	-	-	-	20011	0.45±0.06	0.33-0.58	-	-	13024.48
**Model 2**	20680	4476.4	-4406	-	16892	0.55±0.15	0.24-0.84	0.12±0.05	-	12915.04
**Model 3**	**21223**	**3397.1**	**-4695**	**1486.4**	**16379**	**0.56±0.16**	**0.25-0.86**	**0.09±0.05**	**0.04±0.03**	**12891.84**
**BW52**										
**Model 1**	14937	-	-	-	20496	0.42±0.07	0.28-0.56	-	-	9279.58
**Model 2**	13045	2584.7	-299.32	-	20881	0.36±0.14	0.13-0.66	0.07±0.05	-	9275.76
**Model 3**	**14235**	**2155.7**	**-1086**	**1056.5**	**20185**	**0.39±0.16**	**0.11-0.71**	**0.06±0.04**	**0.03±0.02**	**9263.07**

Table 3: Posterior estimates of variance components and heritability for egg weight and egg production traits using different models

(σ^2_a: Additive genetic variance; σ^2_{mat}: maternal genetic variance; σ_{am}: covariance between additive genetic and maternal genetic effects; σ^2_{mpe}: maternal permanent environmental variance; $h^2_{additive}$ ± S.D.: Posterior direct additive heritability mean ± Posterior standard deviation; $HPDI_{additive}$: Highest Posterior Density Interval for heritability; h^2_{mat} ± S.D.: Posterior maternal heritability mean ± Posterior standard deviation; c^2_{mpe} ± S.D.: Posterior maternal permanent environmental effect mean ± Posterior standard deviation; DIC: Deviance Information Criterion)

Parameters	σ^2_a	σ^2_{mat}	σ_{am}	σ^2_{mpe}	σ^2_e	$h^2_{additive}$ ± S.D.	$HPDI_{additive}$	h^2_{mat} ± S.D.	c^2_{mpe} ± S.D.	DIC
**EW28**										
**Model 1**	2.27	-	-	-	6.92	0.25±0.06	0.13-0.36	-	-	5170.42
**Model 2**	3.08	1.51	-1.54	-	6.23	0.33±0.12	0.13-0.58	0.16±0.05	-	5132.39
**Model 3**	**3.09**	**1.29**	**-1.46**	**0.23**	**6.19**	**0.33±0.12**	**0.12-0.58**	**0.14±0.06**	**0.02±0.02**	**5129.64**
**EW40**										
**Model 1**	3.44	-	-	-	7.43	0.32±0.06	0.19-0.44	-	-	4768.32
**Model 2**	4.29	0.92	-1.01	-	6.84	0.39±0.13	0.13-0.63	0.08±0.05	-	4738.58
**Model 3**	**4.29**	**0.86**	**-1.06**	**0.33**	**6.71**	**0.38±0.13**	**0.12-0.64**	**0.08±0.04**	**0.03±0.02**	**4732.19**
**EW52**										
**Model 1**	8.77	-	-	-	7.63	0.53±0.09	0.36-0.70	-	-	3489.99
**Model 2**	12.29	1.23	-2.35	-	5.81	0.71±0.21	0.32-1.00	0.07±0.05	-	3289.36
**Model 3**	**12.66**	**1.22**	**-2.59**	**0.32**	**5.55**	**0.73±0.22**	**0.29-1.00**	**0.07±0.04**	**0.02±0.02**	**3238.35**
**EP24**										
**Model 1**	10.49	-	-	-	41.2	0.20±0.05	0.11-0.31	-	-	6398.87
**Model 2**	**15.09**	**2.86**	**-4.11**	**-**	**38.64**	**0.29±0.11**	**0.08-0.50**	**0.05±0.03**	**-**	**6385.25**
**Model 3**	13.99	2.49	-3.79	0.96	38.89	0.26±0.12	0.35-0.47	0.05±0.03	0.02±0.02	6389.27
**EP28**										
**Model 1**	20.35	-	-	-	109.98	0.18±0.05	0.09-0.29	-	-	7826.49
**Model 2**	21.35	6.81	-4.82	-	87.85	0.19±0.10	0.02-0.37	0.06±0.04	-	7825.17
**Model 3**	**19.82**	**5.96**	**-5.16**	**3.99**	**86.96**	**0.18±0.10**	**0.05-0.37**	**0.05±0.03**	**0.36±0.25**	**7823.42**
**EP40**										
**Model 1**	69.83	-	-	-	268.15	0.21±0.06	0.10-0.32	-	-	8774.69
**Model 2**	**67.63**	**26.44**	**-15.59**	**-**	**264.1**	**0.19±0.10**	**0.01-0.39**	**0.08±0.04**	**-**	**8773.63**

**Model 3**	62.82	20.22	-14.36	11.51	263.51	0.18±0.09	0.008-0.36	0.06±0.04	0.03±0.03	8773.73
**EP52**										
**Model 1**	60.63	-	-	-	340.66	0.15±0.07	0.04-0.28	-	-	5884.52
**Model 2**	**79.96**	**35.07**	**-34.28**	**-**	**328.77**	**0.19±0.13**	**0.001-0.43**	**0.08±0.05**	**-**	**5881.54**
**Model 3**	71.72	32.88	-33.96	14.81	327.47	0.17±0.12	0.0004-0.41	0.08±0.05	0.03±0.03	5882.44
**EP64**										
**Model 1**	59.5	-	-	-	597.83	0.09±0.06	0.003-0.19	-	-	5615.17
**Model 2**	**33**	**66.61**	**-20.37**	**-**	**589.17**	**0.05±0.05**	**0.0002-0.15**	**0.09±0.05**	**-**	**5614.91**
**Model 3**	36.8	61.41	-25.65	20.74	580.61	0.05±0.05	0.00001-0.16	0.09±0.05	0.03±0.03	5616.3
**EP72**										
**Model 1**	85.78	-	-	-	736.86	0.10±0.06	0.0001-0.22	-	-	5203.88
**Model 2**	62.35	117.6	-48.48	-	704.76	0.07±0.07	0.0006-0.21	0.14±0.07	-	5198.97
**Model 3**	**79.29**	**109.83**	**-67.84**	**32.98**	**689.32**	**0.09±0.07**	**0.0002-0.23**	**0.13±0.07**	**0.04±0.03**	**5198.05**

Posterior genetic and environmental correlations

The posterior means of additive genetic and environmental correlations between all the traits have been presented in Tables 4-6. Additive genetic correlation was positive between all the egg production traits with high and strong positive correlation existing between consecutive traits. For instance, EP24 exhibited high positive additive genetic correlation with EP28 (0.89±0.05) which reduced to almost nil between EP24 and EP72 (0.06±0.25). Traits EP64 and EP72 showed strong and highly positive genetic correlation (0.96±0.02) which indicates that former is a perfect indicator of the overall laying ability of the bird in a cycle. Although EP52 also has high positive correlation with EP72 (0.79±0.11), it is still not enough to choose it as a selection criteria for overall laying ability of the bird. Chandan et al. [40] advocated selecting for higher egg production based on part period egg production of EP52 due to very strong correlation between EP52 and EP64 (0.926±0.050). However, they did not calculate the genetic correlation between EP52 and EP72. Environmental correlation between egg production traits was medium to high between different traits with EP40 and subsequent traits witnessing high correlation values.

Egg weight traits also witnessed very high and positive genetic correlations (>0.90) between the traits. This is in line with the findings by previous workers that genetic correlation between egg weights at different ages was very high, especially between consecutive time periods [71-72]. However, Chandan et al. [40] have obtained comparatively higher estimates between EW28, EW40 and EW52 traits in the same IWH population. This may be due to the reason that our study also included maternal effects in the model which may have partitioned all the genetic parameters amongst different components.

Egg weights were negatively correlated genetically with all the egg production traits with the correlation becoming more strong and negative towards the end of the laying cycle. This is in

agreement with the already known trade-off between egg size and egg production in that consistent selection for higher egg numbers diverts the resources or energy available for producing larger sized eggs [73]. Dam or maternal effect is also an important factor regulating the dynamics of egg production and egg size in females [74]. Since both the set of traits are economically important, it is advised to select for these traits with the help of a selection index according appropriate weightages to all the traits [71].

ASM was negatively correlated genetically with early egg production traits like EP24 (-0.59±0.19), EP28 (-0.84±0.07) and also with EP52 (-0.41±0.20) while it was having a positive correlation with EP64 (0.23±0.28) and EP72 (0.24±0.28). Most of the workers have deduced an antagonistic relationship between ASM and egg production traits [9,75]. The large posterior standard deviation component in the genetic correlation values of ASM with the late EP traits is indicative of increasing the population size to get more accurate genetic picture of relationship between the traits. ASM and BW0 had moderate positive genetic correlation which is obvious given the fact that higher birth weight leads to early sexual maturity due to high positive genetic correlation between the BW0 and body weight at sexual maturity [39,76]. ASM showed positive genetic correlation with the later body weight traits including BW20, BW40 and BW52. It may be on account of the fact that ASM is a trait related to sexual maturity and shares negative correlation with body weight at sexual maturity. However, a positive correlation may exist between ASM and later body weight traits as the late maturing birds have lower egg production and higher body weights due to the existing negative genetic correlation between body weight and production traits [40]. ASM was positively and relatively moderately correlated with all the egg weight traits due to the negative genetic relationship of these traits with egg production as higher ASM will decrease total egg production and ultimately, egg size will increase [73,75].

In agreement with previous studies, there was a strong positive relationship amongst the body weight traits in our study [76]. The correlation between BW0 and rest of the body weight traits was comparatively less than other growth combinations due to the reason that chick birth weight has a strong maternal influence (genetic as well as permanent environmental effect of dam) [58]. However, a negative genetic correlation existed between different egg production traits and body weight traits. This is a reiteration of the oft-repeated remark that lighter hens are more efficient egg producers and that intense selection for egg production introduces severe depression on body weight traits in layers [41,77]. As far as the body weight and egg weight traits were concerned, there was high positive genetic correlation between all the traits. This may be due to obvious physiological reasons and is in line with the observations made by most of the workers [71,78].

The future breeding program of IWH population should be designed considering the additive variability of traits and the genetic correlations obtained in this study. Also, the maternal components of variation should be included along with additive variance for all the economic traits in the BLUP animal model for more accurate estimation. Simultaneously, genetic parameters should be re-estimated after incorporating the data of every completed generation in order to observe the underlying changes in trait variation and also to improve the precision of the genetic estimates.

Table 4: Posterior means of additive genetic and environmental correlation between egg production and egg weight traits

	EP24	EP28	EP40	EP52	EP64	EP72	EW28	EW40	EW52
**EP24**		0.89±0.05	0.50±0.19	0.37±0.21	0.03±0.26	0.06±0.25	-0.36±0.17	-0.34±0.18	-0.51±0.15
**EP28**	0.84±0.01		0.73±0.12	0.43±0.19	0.25±0.24	0.24±0.24	-0.38±0.19	-0.33±0.19	-0.31±0.19
**EP40**	0.39±0.05	0.56±0.03		0.87±0.07	0.76±0.11	0.71±0.13	-0.34±0.18	-0.38±0.17	-0.59±0.14
**EP52**	0.32±0.05	0.47±0.04	0.88±0.01		0.89±0.06	0.79±0.11	-0.54±0.29	-0.84±0.16	-0.74±0.22
**EP64**	0.26±0.06	0.37±0.05	0.76±0.03	0.92±0.01		0.96±0.02	-0.64±0.31	-0.79±0.23	-0.67±0.34
**EP72**	0.21±0.06	0.31±0.05	0.70±0.03	0.86±0.02	0.97±0.004		-0.60±0.33	-0.81±0.19	-0.76±0.25
**EW28**	-0.15±0.06	-0.14±0.06	-0.09±0.05	-0.06±0.06	-0.02±0.07	-0.06±0.07		0.91±0.08	0.91±0.07
**EW40**	-0.46±0.07	-0.06±0.07	-0.09±0.06	-0.05±0.07	-0.08±0.07	-0.05±0.07	0.33±0.06		0.96±0.03
**EW52**	0.04±0.09	-0.09±0.09	0.06±0.09	-0.13±0.09	-0.15±0.10	-0.15±0.09	0.08±0.09	0.27±0.09	

Values above the diagonal indicate posterior additive genetic correlation between the traits whereas values below the diagonal signify posterior environmental correlation between the traits

Table 5: Posterior means of additive genetic and environmental correlation between egg production and body weight traits

	ASM	BW1	BW16	BW20	BW40	BW52	EP24	EP28	EP40	EP52	EP64	EP72
**ASM**		0.32±0.10	-0.12±0.14	0.03±0.14	0.26±0.14	0.32±0.13	-0.59±0.19	-0.84±0.07	0.08±0.29	-0.41±0.20	0.23±0.28	0.24±0.28
**BW1**	-0.54±0.13		0.32±0.09	0.47±0.10	0.37±0.08	0.32±0.09	-0.54±0.19	0.10±0.21	-0.89±0.10	-0.51±0.16	-0.93±0.09	-0.93±0.08
**BW16**	-0.42±0.04	-0.24±0.11		0.89±0.05	0.68±0.09	0.75±0.07	-0.31±0.16	-0.33±0.15	-0.15±0.18	-0.24±0.19	0.08±0.19	0.12±0.19
**BW20**	-0.23±0.05	-0.59±0.12	0.52±0.04		0.87±0.05	0.90±0.03	-0.20±0.17	-0.15±0.17	-0.19±0.18	-0.06±0.21	0.11±0.18	0.14±0.19
**BW40**	-0.09±0.06	-0.44±0.09	0.28±0.06	0.44±0.05		0.96±0.01	-0.28±0.16	-0.35±0.16	-0.15±0.17	-0.32±0.19	0.006±0.18	0.01±0.18
**BW52**	-0.14±0.06	-0.37±0.11	0.25±0.05	0.38±0.05	0.65±0.03		-0.37±0.16	-0.35±0.16	-0.29±0.16	-0.30±0.19	-0.04±0.18	-0.009±0.18
**EP24**	-0.62±0.03	0.27±0.05	0.45±0.06	0.18±0.06	0.02±0.07	0.04±0.07		0.89±0.05	0.50±0.19	0.37±0.21	0.03±0.26	0.06±0.25
**EP28**	-0.49±0.03	-0.12±0.18	0.39±0.05	0.29±0.05	0.12±0.06	0.13±0.07	0.84±0.01		0.73±0.12	0.43±0.19	0.25±0.24	0.24±0.24
**EP40**	-0.19±0.05	0.47±0.05	0.18±0.06	0.15±0.06	0.14±0.07	-0.01±0.08	0.39±0.05	0.56±0.03		0.87±0.07	0.76±0.11	0.71±0.13
**EP52**	-0.28±0.04	0.35±0.15	0.18±0.06	0.15±0.06	0.11±0.07	0.06±0.08	0.32±0.05	0.47±0.04	0.88±0.01		0.89±0.06	0.79±0.11

**EP64**	-0.12±0.05	0.49±0.05	0.07±0.06	0.08±0.07	0.06±0.07	-0.09±0.08	0.26±0.06	0.37±0.05	0.76±0.03	0.92±0.01		0.96±0.02
**EP72**	-0.09±0.05	0.52±0.05	0.05±0.06	0.08±0.07	0.04±0.07	-0.11±0.08	0.21±0.06	0.31±0.05	0.70±0.03	0.86±0.02	0.97±0.004	

Values above the diagonal indicate posterior additive genetic correlation between the traits whereas values below the diagonal signify posterior environmental correlation between the traits

Table 6: Posterior means of additive genetic and environmental correlation between body weight, ASM and egg weight traits

	**ASM**	**BW0**	**BW16**	**BW20**	**BW40**	**BW52**	**EW28**	**EW40**	**EW52**
**ASM**		0.32±0.10	-0.12±0.14	0.03±0.14	0.26±0.14	0.32±0.13	0.32±0.17	0.38±0.15	0.17±0.17
**BW0**	-0.54±0.13		0.32±0.09	0.47±0.10	0.37±0.08	0.32±0.09	0.79±0.19	0.86±0.14	0.86±0.14
**BW16**	-0.42±0.04	-0.24±0.11		0.89±0.05	0.68±0.09	0.75±0.07	0.49±0.13	0.54±0.11	0.77±0.09
**BW20**	-0.23±0.05	-0.59±0.12	0.52±0.04		0.87±0.05	0.90±0.03	0.58±0.13	0.73±0.15	0.65±0.11
**BW40**	-0.09±0.06	-0.44±0.09	0.28±0.06	0.44±0.05		0.96±0.01	0.59±0.11	0.72±0.07	0.61±0.09
**BW52**	-0.14±0.06	-0.37±0.11	0.25±0.05	0.38±0.05	0.65±0.03		0.37±0.14	0.62±0.09	0.57±0.11
**EW28**	0.06±0.05	-0.18±0.06	-0.02±0.06	0.08±0.07	0.13±0.07	0.13±0.07		0.91±0.08	0.91±0.07
**EW40**	-0.02±0.06	-0.49±0.04	-0.08±0.07	-0.25±0.09	-0.009±0.08	-0.009±0.09	0.33±0.06		0.96±0.03
**EW52**	-0.04±0.08	-0.93±0.009	-0.25±0.11	-0.06±0.12	-0.09±0.12	-0.01±0.15	0.08±0.09	0.27±0.09	

Values above the diagonal indicate posterior additive genetic correlation between the traits whereas values below the diagonal signify posterior environmental correlation between the traits

Conclusions

This study was conducted to delineate the role of maternal genetic and permanent environment in variance partitioning and genetic parameters of various economic traits in a population of White Leghorn layer line IWH using Bayesian approach. Most of the economic traits of the population are affected by maternal effects, though the variance attributable to maternal genetic and permanent environmental effects was low (<0.1) for most of the traits. EW28 and EP72 exhibited comparatively higher values for maternal h^2 while maternal permanent environmental effect was quite high for the trait EP28. Although using Bayesian inference improved the accuracy of additive genetic variance and direct heritability estimates, smaller population size was still a limitation in getting precise estimates for maternal components of variance. Maternal variance estimates can be improved upon by increasing the data size of the population and further evaluation and fitting of the models using Bayesian methods.

Acknowledgements

The authors are thankful to the Director, ICAR-Directorate of Poultry Research, Hyderabad for his constant support and encouragement. The technical services provided by the farm and hatchery staffs are duly acknowledged.

Author contributions

Conceptualization- Aneet Kour, U Rajkumar, RN Chatterjee

Data curation- Aneet Kour, K.S. Rajaravindra, M Niranjan

Analysis- Aneet Kour, L. Leslie Leo Prince, Santosh Haunshi

Interpretation- U Rajkumar, M. Niranjan, Santosh Haunshi, BLN Reddy

Manuscript writing- Aneet Kour, K.S. Rajaravindra

Manuscript review and editing- L. Leslie Leo Prince, BLN Reddy, U Rajkumar, RN Chatterjee

Data availability statement

Data associated with this study is a property of Indian Council of Agricultural Research (ICAR) and can be made available upon reasonable request to ICAR-DPR, Hyderabad. We are also in the process of submitting the data in ICAR research data repository for knowledge management and URL for same will be provided in short course of time.

Conflict of Interest

All the authors declare that they have no financial or other interests which could be construed as potential conflict of interest.

References

1. Tan X, Liu R, Li W, Zheng M, Zhu D, Liu D, et al. Assessment the effect of genomic selection and detection of selective signature in broilers. Poult Sci. 2022;101(6):101856. https://doi.org/10.1016/j.psj.2022.101856
2. Korver DR. Review: Current challenges in poultry nutrition, health, and welfare. animal. 2023;17:100755. https://doi.org/10.1016/j.animal.2023.100755
3. Bulmer MG. The Effect of Selection on Genetic Variability. The American Naturalist. 1971;105(943):201–11. https://www.journals.uchicago.edu/doi/10.1086/282718
4. Falconer DS, Mackay TFC. Introduction to Quantitative Genetics. 4th ed. Addison Wesley Longman, Harlow; 1996
5. Sosa-Madrid BS, Maniatis G, Ibáñez-Escriche N, Avendaño S, Kranis A. Genetic Variance Estimation over Time in Broiler Breeding Programmes for Growth and Reproductive Traits. Animals. 2023;13(21):3306. **https://doi.org/10.3390/ani13213306**

6. Mayhew AJ, Meyre D. Assessing the Heritability of Complex Traits in Humans: Methodological Challenges and Opportunities. Curr Genomics. 2017;18(4):332. https://doi.org/10.2174/1389202918666170307161450
7. Worku D, Gowane G, Alex R, Joshi P, Verma A. Inputs for optimizing selection platform for milk production traits of dairy Sahiwal cattle. PLoS One. 2022;17(5):e0267800. https://doi.org/10.1371/journal.pone.0267800
8. Grosso JLBM, Balieiro JCC, Eler JP, Ferraz JBS, Mattos EC, Filho TM. Comparison of different models to estimate genetic parameters for carcass traits in a commercial broiler line. Genet Mol Res. 2010;9(2):908–18. https://doi.org/10.4238/vol9-2gmr773
9. Prince LLL, Rajaravindra KS, Rajkumar U, Reddy BLN, Paswan C, Haunshi S, et al. Genetic analysis of growth and egg production traits in synthetic colored broiler female line using animal model. Trop Anim Health Prod. 2020;52(6):3153–63. https://doi.org/10.1007/s11250-020-02340-4
10. Rajkumar U, Leslie Leo Prince L, Rajaravindra KS, Haunshi S, Niranjan M, Chatterjee RN. Analysis of (co) variance components and estimation of breeding value of growth and production traits in Dahlem Red chicken using pedigree relationship in an animal model. PLoS One. 2021;16(3):e0247779. https://doi.org/10.1371/journal.pone.0247779
11. Waldron DF, Morris CA, Baker RL, Johnson DL. Maternal effects for growth traits in beef cattle. Livest Prod Sci. 1993 Mar 1;34(1–2):57–70. https://doi.org/10.1016/0301-6226(93)90035-G
12. Bijma P. Estimating maternal genetic effects in livestock. J Anim Sci. 2006;84(4):800–6. https://doi.org/10.2527/2006.844800x

13. Liu C, Dupuis J, Larson MG, Cupples LA, Ordovas JM, Vasan RS, et al. Revisiting heritability accounting for shared environmental effects and maternal inheritance. Hum Genet. 2015;134(2):169–79. https://doi.org/10.1007/s00439-014-1505-6
14. Rubolini D, Romano M, Navara KJ, Karadas F, Ambrosini R, Caprioli M, et al. Maternal effects mediated by egg quality in the Yellow-legged Gull Larus michahellis in relation to laying order and embryo sex. Front Zool. 2011;8(1):1–15. https://doi.org/10.1186/1742-9994-8-24
15. Abou Khadiga G, Mahmoud BYF, El-Full EA. Genetic evaluation of early egg production and maturation traits using two different approaches in Japanese quail. Poult Sci. 2016;95(4):774–9. https://doi.org/10.3382/ps/pev386
16. Jasouri M, Zamani P, Alijani S. Dominance genetic and maternal effects for genetic evaluation of egg production traits in dual-purpose chickens. Br Poult Sci. 2017;58(5):498–505. https://doi.org/10.1080/00071668.2017.1336748
17. Sarvari-Kalouti H, Maghsoudi A, Rokouei M, Faraji-Arough H, Bagherzadeh-Kasmani F. Direct and maternal genetic effects for preinflection point growth traits and humoral immunity in quail. Poult Sci. 2023;102(2):102340. https://doi.org/10.1016/j.psj.2022.102340
18. Hartmann C, Johansson K, Strandberg E, Rydhmer L. Genetic correlations between the maternal genetic effect on chick weight and the direct genetic effects on egg composition traits in a White Leghorn line. Poult Sci. 2003;82(1):1–8. https://doi.org/10.1093/ps/82.1.1
19. Kruuk LEB, Hadfield JD. How to separate genetic and environmental causes of similarity between relatives. J Evol Biol. 2007;20(5):1890–903. https://doi.org/10.1111/j.1420-9101.2007.01377.x

20. Ullengala R, Prince LLL, Paswan C, Haunshi S, Chatterjee R. Variance component analysis of growth and production traits in Vanaraja male line chickens using animal model. Anim Biosci. 2021;34(4):471. https://doi.org/10.5713/ajas.19.0826
21. Kemper KE, Goddard ME. Understanding and predicting complex traits: knowledge from cattle. Hum Mol Genet. 2012;21(R1):R45–51. https://doi.org/10.1093/hmg/dds332
22. Zhang J, Schumacher FR. Evaluating the estimation of genetic correlation and heritability using summary statistics. Mol Genet Genomics. 2021;296(6):1221. https://doi.org/10.1007/s00438-021-01817-7
23. Yadav N, Mukherjee S, Mukherjee A. Comparative genetic analysis of frequentist and Bayesian approach for reproduction, production and life time traits showing favourable association of age at first calving in Tharparkar cattle. Anim Biosci. 2023;36(12):1806. https://doi.org/10.5713/ab.23.0025
24. McNeish D. On using Bayesian methods to address small sample problems. Structural Equation Modeling: A Multidisciplinary Journal. 2016;23(5):750-73. https://doi.org/10.1080/10705511.2016.1186549
25. van de Schoot R, Depaoli S, King R, Kramer B, Märtens K, Tadesse MG, et al. Bayesian statistics and modelling. Nat Rev Methods Prim 2021. 2021 1(1):1–26. https://doi.org/10.1038/s43586-020-00001-2
26. Wolc A, Stricker C, Arango J, Settar P, Fulton JE, O'Sullivan NP, et al. Breeding value prediction for production traits in layer chickens using pedigree or genomic relationships in a reduced animal model. Genet Sel Evol. 2011;43(1):1–9. https://doi.org/10.1186/1297-9686-43-5

27. Yousefi Zonuz A, Alijani S, Mohammadi H. Estimation of Genetic Parameters for Production and Reproduction Traits of Iranian Native Chickens using Bayesian Method Via Gibbs Sampling. Res Anim Prod. 2014;4(8):91–9.

28. Mohammadi A, Naderi Y, Nabavi R, Jafari F. Determination of the best model for estimation of genetic parameters on the Fars native chicken traits using Bayesian and REML methods. Genetika. 2018;50(2):431–47. https://doi.org/10.2298/GENSR1802431M

29. Misztal I, Tsuruta S, Lourenco D A L, Masuda Y, Aguilar I, Legarra A, Vitezica Z Manual for BLUPF90 family programs. University of Georgia. 2018. http://nce.ads.uga.edu/wiki/doku.php?id=documentation

30. Kour A, Deb SM, Nayee N, Niranjan SK, Raina VS, Mukherjee A, et al. Novel insights into genome-wide associations in Bos indicus reveal genetic linkages between fertility and growth. Anim Biotechnol. 2023;34(1):39–55. https://doi.org/10.1080/10495398.2021.1932520

31. Spiegelhalter DJ, Best NG, Carlin BP, Van Der Linde A. Bayesian Measures of Model Complexity and Fit. J R Stat Soc Ser B Stat Methodol. 2002;64(4):583–639. https://dx.doi.org/10.1111/1467-9868.00353

32. Tongsiri S, Jeyaruban MG, van der Werf JHJ. Genetic parameters for egg production traits in purebred and hybrid chicken in a tropical environment. Br Poult Sci. 2015;56(6):613–20. https://doi.org/10.1080/00071668.2015.1099614

33. Kang H, Ren M, Li S, Lu Y, Deng X, Zhang Z, et al. Estimation of genetic parameters for important traits using a multi-trait model in late-feathering Qingyuan partridge hens in China. J Anim Breed Genet. 2023;140(2):158–66. https://doi.org/10.1111/jbg.12739

34. Chu TT, Madsen P, Norberg E, Wang L, Marois D, Henshall J, et al. Genetic analysis on body weight at different ages in broiler chicken raised in commercial environment. J Anim Breed Genet. 2020;137(2):245–59. https://doi.org/10.1111/jbg.12448

35. Mrode R A, Thompson R. Linear models for the prediction of Animal Breeding Values. CABI Publishing, UK; 2005.

36. Harry ND, Zakas C. Maternal patterns of inheritance alter transcript expression in eggs. BMC Genomics. 2023;24(1):1–13. https://doi.org/10.1186/s12864-023-09291-8

37. Kang L, Zhang Y, Zhang N, Zang L, Wang M, Cui X, et al. Identification of differentially expressed genes in ovaries of chicken attaining sexual maturity at different ages. Mol Biol Rep. 2012;39(3):3037–45. https://doi.org/10.1007/s11033-011-1066-x

38. Wright D, Rubin C, Schutz K, Kerje S, Kindmark A, Brandström H, et al. Onset of Sexual Maturity in Female Chickens is Genetically Linked to Loci Associated with Fecundity and a Sexual Ornament. Reprod Domest Anim. 2012;47(SUPPL. 1):31–6. https://doi.org/10.1111/j.1439-0531.2011.01963.x

39. Soller M, Brody T, Eitan Y, Agursky T, Wexler C. Minimum Weight for Onset of Sexual Maturity in Female Chickens: Heritability and Phenotypic and Genetic Correlations with Early Growth Rate. Poult Sci. 1984;63(11):2103–13. https://doi.org/10.3382/ps.0632103

40. Chandan P, Prince LLL, Bhattacharya TK, Rajkumar U, Chatterjee RN. Estimation of heritability and genetic correlation of egg production traits using animal model in commercial layer. Indian J Anim Sci. 2019a;89(11):1269–73.

41. Bish CL, BEANE WL, RUSZLER PL, CHERRY JA. Body Weight Influence on Egg Production. Poult Sci. 1985;64(12):2259–62. https://doi.org/10.3382/ps.0642259

42. Lacin E, Yildiz A, Esenbuga N, Macit M. Effects of differences in the initial body weight of groups on laying performance and egg quality parameters of Lohmann laying hens. Czech J Anim Sci. 2008;53(11):466–71.
43. Kirkpatrick M, Lande R. The evolution of maternal characters. Evolution. 1989 May 1;43(3):485-503.
44. Celeux G, Forbesy F, Robertz CP, Titteringtonx DM. Deviance information criteria for missing data models. Bayesian Anal. 2006;1(4): 651 - 73. https://doi.org/10.1214/06-BA122
45. Plummer M. Penalized loss functions for Bayesian model comparison. Biostatistics. 2008;9(3):523–39. https://doi.org/10.1093/biostatistics/kxm049
46. Pooley CM, Marion G. Bayesian model evidence as a practical alternative to deviance information criterion. R Soc Open Sci. 2018;5(3). **https://doi.org/10.1098/rsos.171519**
47. Manjeet, Dalal DS, Dahiya SP, Patil CS. Genetic parameters of various performance traits in different generations of synthetic White Leghorn strain. International Journal of Advanced Biological Research. 2018; 8(4):438-40.
48. Mucha S, Wolc A, Szwaczkowski T. Bayesian and REML analysis of twinning and fertility in Thoroughbred horses. Livest Sci. 2012;144(1–2):82–8. https://doi.org/10.1016/j.livsci.2011.10.015
49. Grantham KL, Kasza J, Heritier S, Carlin JB, Forbes AB. Evaluating the performance of Bayesian and restricted maximum likelihood estimation for stepped wedge cluster randomized trials with a small number of clusters. BMC Med Res Methodol. 2022;22(1):1–18. https://doi.org/10.1186/s12874-022-01550-8

50. Barot VN, Savaliya FP, Hirani ND, Patel AB, Vataliya PH, Khanna K, et al. Genetic parameters of various economic traits in different generations of synthetic White Leghorn. Indian J Poult Sci. 2008;43(1):20–4.
51. Chandan P, Bhattacharya TK, Rajkumar U, Prince LLL, Chatterjee RN. Estimation of genetic parameters of growth and egg production traits by animal model in IWK layer strain. Indian J Anim Res. 2019b;53(9):1252-7. https://doi.10.18805/ijar.B-3638
52. Rosa JO, Venturini GC, Chud TCS, Pires BC, Buzanskas ME, Stafuzza NB, et al. Bayesian inference of genetic parameters for reproductive and performance traits in white leghorn hens. Czech J Anim Sci. 2018;63(6):230–6. https://doi.10.17221/116/2017-CJAS
53. Laxmi P J, Gupta B R, Chatterjee R N, Sharma R P, Reddy V R. Genetic analysis of growth and production traits in IWK strain of White Leghorn. Ind J Poult Sci. 2010; 45(2):123-6.
54. Sreenivas D, Prakash M G, Chatterjee R N, Mahender M. Genetic analysis of productive and reproductive traits in White Leghorn chicken. Ind J Poult Sci. 2012;47(3):274-80.
55. Du Plessis PHC, Erasmus J. The relationship between egg production, egg weight and body weight in laying hens. Worlds Poult Sci J. 1972;28(3):301–10.
56. Şekeroğlu A, Altuntaş E. Effects of egg weight on egg quality characteristics. J Sci Food Agric. 2009;89(3):379–83. **https://doi.org/10.1002/jsfa.3454**
57. Alkan S, Galiç A, Karsli T, Karabaɫ K. Effects of egg weight on egg quality traits in partridge (Alectoris Chukar). J Appl Anim Res. 2015;43(4):450–6. https://doi.org/10.1080/09712119.2014.980419
58. Hartmann C, Strandberg E, Rydhmer L, Johansson K. Genetic Relations between Reproduction, Chick Weight and Maternal Egg Composition in a White Leghorn Line.

Acta Agric Scand Sect A - Anim Sci. 2002;52(2):91–101. https://doi.org/10.1080/09064700212072

59. Pick JL, Ebneter C, Hutter P, Tschirren B. Disentangling Genetic and Prenatal Maternal Effects on Offspring Size and Survival. Am Nat. 2016;188(6):628–39. https://doi.10.1086/688918

60. Chatterjee RN, Niranjan M, Panda AK. Inheritance of important economic traits in 3 pure lines and a control population of White Leghorn. Indian J Anim Sci. 2008;78(1):75-9.

61. Veeramani P, Churchil RR, Kutty KN. Estimates of heritability and correlations of economic traits in two strains of White Leghorn. International Journal of Veterinary Science. 2012;1(2):45-8.

62. Dash SK, Gupta AK, Singh A, Chakravarty AK, Madhusoodanan M, Valsalan J, et al. Evaluation of efficiency of sire model and animal model in Holstein Friesian crossbred cattle considering first lactation production and fertility traits. Vet World. 2014; 7(11). https://doi.10.14202/vetworld.2014.933-937

63. Haunshi S, Padhi MK, Chatterjee RN, Bhattacharya TK, Rajaravindra KS. Genetic characterization of layer germplasm evolved by AICRP on poultry breeding. Indian J Anim Sci. 2016; 86(12):1431-5.

64. Wolc A, Szwaczkowski T. Estimation of genetic parameters for monthly egg production in laying hens based on random regression models. J Appl Genet. 2009;50(1):41–6. https://doi.org/10.1007/BF03195650

65. Venturini GC, Grossi DA, Ramos SB, Cruz VAR, Souza CG, Ledur MC, et al. Estimation of genetic parameters for partial egg production periods by means of random regression models. Genet Mol Res. 2012;11(3):1819–29. https://doi.org/10.4238/2012.July.10.18

66. Chomchuen K, Tuntiyasawasdikul V, Chankitisakul V, Boonkum W. Genetic Evaluation of Body Weights and Egg Production Traits Using a Multi-Trait Animal Model and Selection Index in Thai Native Synthetic Chickens (Kaimook e-san2). Animal. 2022;12(3):335. https://doi.org/10.3390/ani12030335

67. Schaeffer LR. Application of random regression models in animal breeding. Livest Prod Sci. 2004;86(1–3):35–45. https://doi.org/10.1016/S0301-6226(03)00151-9

68. Marshall DJ, Uller T. When is a maternal effect adaptive? Oikos. 2007;116(12):1957–63. **https://doi.org/10.1111/j.2007.0030-1299.16203.x**

69. Widowski TM, Cooley L, Hendriksen S, Peixoto MRLV. Maternal age and maternal environment affect egg composition, yolk testosterone, offspring growth and behaviour in laying hens. Sci Reports. 2022;12(1):1–12. https://doi.org/10.1038/s41598-022-05491-6

70. Romé H, Chu TT, Marois D, Huang CH, Madsen P, Jensen J. Estimation and consequences of direct-maternal genetic and environmental covariances in models for genetic evaluation in broilers. Genet Sel Evol. 2023;55(1):1–15. https://doi.org/10.1186/s12711-023-00829-8

71. Niknafs S, Nejati-Javaremi A, Mehrabani-Yeganeh H, Fatemi SA. Estimation of genetic parameters for body weight and egg production traits in Mazandaran native chicken. Trop Anim Health Prod. 2012;44(7):1437–43. https://doi.org/10.1007/s11250-012-0084-6

72. Ni A, Calus MPL, Bovenhuis H, Yuan J, Wang Y, Sun Y, et al. Genetic parameters, reciprocal cross differences, and age-related heterosis of egg-laying performance in chickens. Genet Sel Evol. 2023;55(1):1–18. https://doi.org/10.1186/s12711-023-00862-7

73. Smalås A, Amundsen PA, Knudsen R. The trade-off between fecundity and egg size in a polymorphic population of Arctic charr (Salvelinus alpinus (L.)) in Skogsfjordvatn, subarctic Norway. Ecol Evol. 2017;7(7):2018–24. **https://doi.org/10.1002/ece3.2669**

74. Wootton RJ. Ecology of Teleost Fishes. Ecol Teleost Fishes. 2nd ed. Springer, Netherlands; 1998.

75. Akbas Y, Takma, CH. Canonical correlation analysis for studying the relationship between egg production traits and body weight, egg weight and age at sexual maturity in layers. Czech Journal of Animal Science, 2005;50(4):163-8.

76. Dana N, vander Waaij EH, van Arendonk JAM. Genetic and phenotypic parameter estimates for body weights and egg production in Horro chicken of Ethiopia. Trop Anim Health Prod. 2011;43(1):21–8. https://doi.org/10.1007/s11250-010-9649-4

77. Lerner IM. Natural Selection and Egg Size in Poultry. AM Nat. 1951;85(825):365–72.

78. Lin RL, Chen HP, Rouvier R, Marie-Etancelin C. Genetic parameters of body weight, egg production, and shell quality traits in the Shan Ma laying duck (Anas platyrhynchos). Poult Sci. 2016;95(11):2514–9. https://doi.org/10.3382/ps/pew222
